# Metastatic colorectal cancer responsive to regorafenib for 2 years: a case report

**DOI:** 10.1186/s13256-017-1366-4

**Published:** 2017-08-18

**Authors:** Kenji Yoshino, Dai Manaka, Ryo Kudo, Shunpei Kanai, Eisei Mitsuoka, Satoshi Kanto, Shinya Hamasu, Sayuri Konishi, Ryuta Nishitai

**Affiliations:** Department of Surgery, Kyoto Katsura Hospital, 17, Yamada Hirao-cho, Nishikyo-ku, Kyoto-city, Kyoto 615-8256 Japan

**Keywords:** Colorectal cancer, Regorafenib, Lymph node metastasis, Partial response

## Abstract

**Background:**

Regorafenib is an oral multikinase inhibitor that has been demonstrated as clinically effective in patients with metastatic colorectal cancer in phase III studies. Although disease control was achieved in 40% of the pretreated patients with metastatic colorectal cancer in the pivotal studies, radiological response has rarely been reported. Severe adverse events associated with regorafenib are known to occur during the first and second courses of treatment. We present a case of a 62-year-old Japanese patient whose metastatic colorectal cancer has been responding to treatment with regorafenib for 2 years.

**Case presentation:**

A 54-year-old Japanese man visited our institute exhibiting general malaise, and he was diagnosed with ascending colon cancer in April 2006. He underwent right hemicolectomy, and the final staging was T3N0M0, stage II. After 19 months, pulmonary metastasis and anastomotic recurrences were detected, and a series of operations were performed to resect both metastatic lesions. After that, liver metastasis, a duodenal metastasis with right renal invasion, right adrenal metastasis, and para-aortic lymph node metastases were observed during follow-up, and chemotherapy and resection were performed. The patient had metastatic para-aortic lymph nodes after the fifth tumor resection and underwent multiple lines of chemotherapy in April 2014. Regorafenib monotherapy was started at 80 mg/day. Then, regorafenib was increased to 120 mg/day in the second cycle. Regorafenib monotherapy led to 60% tumor shrinkage within the initial 2 months, and the tumor further decreased in size over 4 months until it became unrecognizable on imaging studies. The clinical effects of regorafenib monotherapy have shown a partial response according to Response Evaluation Criteria in Solid Tumors criteria. No severe adverse events were observed, except for mild fatigue and hand-foot syndrome. The patient has received 24 courses of regorafenib over 2 years without exhibiting tumor progression.

**Conclusions:**

To the best of our knowledge, this is the longest treatment with regorafenib without tumor progression ever reported. A reduced dosage of regorafenib at induction may ameliorate the cutaneous and hepatic toxicity associated with its use.

## Background

A pivotal phase III clinical trial, the Colorectal Cancer Treated with Regorafenib or Placebo after Failure of Standard Therapy (CORRECT) study, demonstrated that regorafenib reduces disease progression and prolongs median survival in patients with metastatic colorectal cancer (mCRC) [1]. Whereas disease control was achieved in 41% of the patients, objective response was observed in only 1% of the regorafenib recipients in the study. There have been few publications describing the detailed clinical course of patients with mCRC respondent to regorafenib [2, 3], and the longest duration of treatment with regorafenib in the phase III study was 16 months, which is the longest treatment ever reported. Therefore, it is important to accumulate information on the clinical effects of regorafenib. We report a case of a patient with mCRC who has responded to regorafenib for 24 months.

## Case presentation

**Fig. 1:**
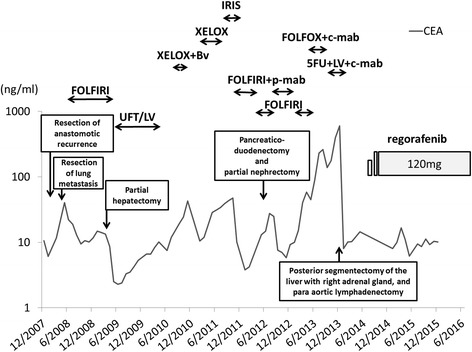
Summary of the treatments. Trends of carcinoembryonic antigen levels is shown by *solid* *lines*. Low level of the tumor markers has been maintained throughout the treatment with regorafenib. *CEA* Carcinoembryonic antigen, *FOLFIRI* Folinic acid, fluorouracil, and irinotecan therapy, *XELOX* Capecitabine and oxaliplatin, *Bv* Bevacizumab, *c-mab* Cetuximab, *LV* Leucovorin, *FU* Fluorouracil, *p-mab* Panitumumab, *IRIS* ​irinotecan and S-1, *UFT *tegafur and uracil

A 54-year-old Japanese man visited our institute exhibiting general malaise in April 2006, and he was diagnosed with ascending colon cancer. He underwent right hemicolectomy, and the pathological diagnosis was moderately differentiated tubular adenocarcinoma with vascular involvement (proximal and distal margin >10 cm). An intraoperative frozen section was not obtained. The final staging was T3N0M0, stage II, according to the TNM classification of the Union for International Cancer Control, seventh edition [4]. The patient did not receive adjuvant chemotherapy according to the Japanese guidelines [5]. The patient’s progress after the first operation is shown in Fig. 1.

In December 2007, after 20 months of periodic follow-up, the patient complained of abdominal distention lasting for about 1 month. Contrast-enhanced computed tomography (CECT) demonstrated pulmonary and anastomotic recurrences, and a series of operations were performed to resect both metastatic lesions (partial pulmonary and anastomotic resection). Immediately after resection of the lung metastasis, a small liver metastasis (10 mm) in the left medial section and para-aortic lymph node metastases (18 mm) were found. First-line chemotherapy was initiated using a doublet regimen of fluorouracil (FU) and irinotecan therapy (FOLFIRI). The para-aortic lymph nodes were extinguished by 23 courses of FOLFIRI, and the fourth operation was performed to resect the liver metastasis (partial resection). Oral FU and leucovorin (LV) were administered for 1 year as adjuvant chemotherapy. Six months later, in October 2010, periodic CECT demonstrated a duodenal metastasis with right renal invasion. Capecitabine and oxaliplatin (XELOX) with bevacizumab (B-mab) were started as the second-line chemotherapy, but this was interrupted by a sigmoid colon perforation during the third course (fifth operation; sigmoidectomy). There was no malignancy in the perforated sigmoid area. The patient received six additional courses of XELOX without B-mab to avoid further risk of gastrointestinal perforation [6]. With the tumor progression, FOLFIRI was rechallenged (third-line chemotherapy) with the anti-epidermal growth factor receptor antibody panitumumab (P-mab). The tumor responded to FOLFIRI/P-mab. After eight courses of treatment, a pancreaticoduodenectomy with partial right nephrectomy was performed in March 2012 (sixth operation).

In July 2012, 4 months after the sixth surgery, right adrenal and para-aortic lymph node metastases were observed. These lesions did not respond to FOLFIRI/P-mab. The fourth-line chemotherapy, including oxaliplatin (mFOLFOX6: folinic acid, fluorouracil, and oxaliplatin) plus P-mab or cetuximab (C-mab), was discontinued because of grade 2 adverse events (AEs), including allergy accompanied by rash to oxaliplatin (Common Terminology Criteria for Adverse Events [CTCAE] version 4.0 [7]). The tumor progressed during further treatment with FU/LV/C-mab. The seventh operation was performed in December 2013 to resect the right adrenal gland, right posterior section of the liver, inferior vena cava, and para-aortic lymph nodes.

**Fig. 2:**
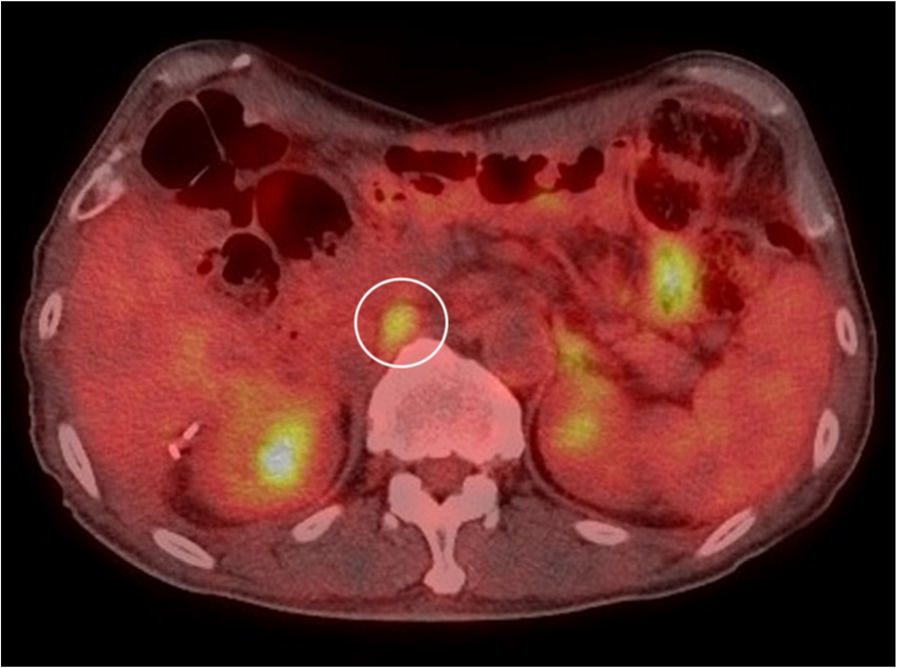
Lymph node metastasis. ﻿The circle indicate﻿s high standardized uptake value of the paracaval lymph node that was observed by ^18^F-fluorodeoxyglucose positron-emission tomography/computed tomography before the beginning of treatment with regorafenib

**Fig. 3:**
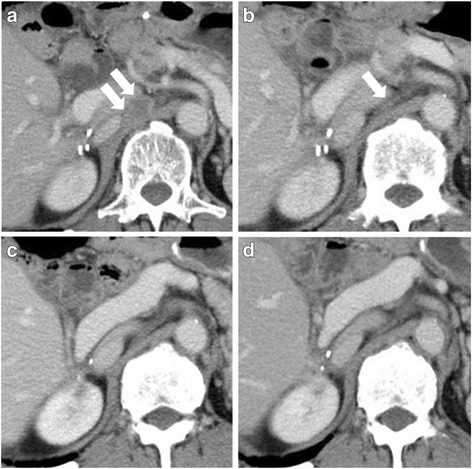
Computed tomographic images of the metastatic lymph node during the time course of treatment with regorafenib. The *arrows* indicate the metastatic lymph node.﻿ **a** Paracaval metastatic lymph node before the beginning of regorafenib therapy. **b** Metastatic lymph node decreased in size after 4 months of regorafenib therapy. **c** Metastatic lymph node became indistinguishable after 9 months of regorafenib treatment. **d** Tumor response was maintained 25 months after the beginning of regorafenib treatment

Paracaval lymph node metastasis was detected by ^18^F-fluorodeoxyglucose positron emission tomography/computed tomography (^18^F-FDG PET/CT) (maximum standardized uptake value 3.8) in April 2014 (Fig. 2). Chemotherapy could not be started immediately, because severe general malaise had deteriorated the patient’s compliance (performance score 2 according to the Eastern Cooperative Oncology Group Scale of Performance Status). The metastatic lesions had enlarged during the time course (Fig. 3a). In June 2014, regorafenib was administered at 80 mg once daily for 3 weeks in the initial month, and we followed the patient as an outpatient once per week. Confirming there were no AEs except grade 1 fatigue and hand-foot syndrome, regorafenib was increased to 120 mg in the second cycle. Two months later, the metastatic lymph node had shrunk by approximately 60% (Fig. 3b), and the lesion further decreased in size throughout the following 4 months, until it had almost vanished as visualized by CECT (Fig. 3c). One of the tumor markers, carcinoembryonic antigen (CEA), was beyond the normal range and stayed between 6.2 and 16.7 ng/ml, although it did not correlate with the tumor volume (Fig. 3d). The clinical effects of regorafenib monotherapy were classified as partial response according to Response Evaluation Criteria in Solid Tumors (RECIST) version 1.1 criteria [8], and the patient has currently received 24 courses of regorafenib over 2 years without exhibiting tumor progression. CECT was used for periodic screening, and ^18^F-FDG PET/CT was performed once, but metastasis could not be identified. The dose of regorafenib was fixed to 120 mg/day and could not be escalated to the full 160 mg/day, owing to several AEs. Among the frequent AEs, such as hypertension, hand-foot syndrome, diarrhea, fatigue, stomatitis, and hoarseness, the only grade 3 AE was proteinuria.

## Discussion

Regorafenib reduced the risk of progression of pretreated patients with mCRC by 51–69% and prolonged overall survival for 1.4–2.5 months in phase III studies [1, 9]. Although this drug had a high disease control rate of 41–51% for patients with mCRC, radiological response has rarely been observed. Response as defined by the RECIST version 1.1 criteria occurred in only 1.0–4.4% of regorafenib recipients in previous studies. There has been only one paper published describing radiological response of lung metastases with regorafenib treatment [2].

The case of our patient demonstrates complete response of the targeted paracaval lymph node. This case was classified as a partial response by RECIST criteria because the patient’s CEA level remained above the normal limit. Periodic radiological screening has not revealed any lesion to account for the abnormal tumor marker, and this case has been clinically considered as complete remission. The longest duration of regorafenib treatment in the phase III study was 16 months [9], which is the longest treatment ever reported. Given that there were no signs of viable tumor after 24 courses of treatment in our patient, there may be an option to withhold drug administration until disease progression.

Prolonged treatment with regorafenib in our patient may be partly attributable to the mild AEs. Severe AEs associated with regorafenib are known to occur during the first and second courses of treatment [10]. Although our patient had a deteriorated health condition at the beginning of this treatment, regorafenib did not lead to severe AEs. The standard dose of regorafenib is 160 mg/day. In cases of AEs, the dose is reduced to 120 mg/day. Recent results from Japanese postmarketing surveillance suggest a possibility that induction of regorafenib at a reduced dose may attenuate cutaneous and hepatic toxicity [11]. It is noteworthy that the reduced dose did not seem to impair the antitumor power in our patient.

## Conclusions

We describe a case of a patient with mCRC who showed a partial response to treatment with regorafenib after receiving multiple lines of chemotherapy. Induction of regorafenib at a reduced dose may attenuate severe AEs. Dose escalation may be an option if the patient cannot tolerate the standard dose reduction protocol.

## Abbreviations

AE: Adverse event
B-mab: Bevacizumab
CEA: Carcinoembryonic antigen
CECT: Contrast-enhanced computed tomography
C-mab: Cetuximab
CORRECT: Colorectal Cancer Treated with Regorafenib or Placebo after Failure of Standard Therapy study
CTCAE: Common Terminology Criteria for Adverse Events
^18^F-FDG PET/CT: ^18^F-fluorodeoxyglucose positron-emission tomography/computed tomography
FOLFIRI: Fo﻿linic acid, fluorouracil, and irinotecan therapy
FU: Fluorouracil
LV: Leucovorin
mCRC: Metastatic colorectal cancer
P-mab: Panitumumab
RECIST: Response Evaluation Criteria in Solid Tumors
XELOX: Capecitabine and oxaliplatin
